# *MIF* -173 G > C (rs755622) Gene Polymorphism Modulates Tuberculosis Risk: Evidence from a Meta-analysis and Trial Sequential Analysis

**DOI:** 10.1038/s41598-017-17308-y

**Published:** 2017-12-05

**Authors:** Mohammed Y. Areeshi, Raju K. Mandal, Sajad A. Dar, Arshad Jawed, Mohd Wahid, Mohtashim Lohani, Aditya K. Panda, B. N. Mishra, Naseem Akhter, Shafiul Haque

**Affiliations:** 1 Research and Scientific Studies Unit, College of Nursing & Allied Health Sciences, Jazan University, Jazan, 45142 Saudi Arabia; 2 University College of Medical Sciences & GTB Hospital (University of Delhi), Delhi, 110095 India; 3 Centre for Life Sciences, Central University of Jharkhand, Ranchi, 835205 Jharkhand India; 4 Department of Biotechnology, Institute of Engineering & Technology, Lucknow, 226021 Uttar Pradesh India; 5 Department of Laboratory Medicine, Faculty of Applied Medical Sciences, Albaha University, Albaha, 65431 Saudi Arabia

## Abstract

The macrophage migration inhibitory factor (MIF) is a cytokine that plays an important role in inhibiting the growth of pathogenic *Mycobacterium tuberculosis* (*M.tb*) and regulates immune responses against *M.tb* pathogen. *MIF* -173 G > C gene polymorphism may affect immunity in an individual and leads to susceptibility to tuberculosis (TB). A large number of studies have investigated the relevance of this polymorphism with TB risk, but their results were inconclusive. To obtain a precise conclusion, a meta-analysis was performed by retrieving six eligible studies from Google Scholar, PubMed (Medline), and EMBASE online databases. Overall combined analysis suggested increased TB risk between *MIF* -173 G > C polymorphism and overall risk in four genetic models, i.e., allelic (C vs. G: p = 0.001; OR = 1.517, 95% CI = 1.312 to 1.753), homozygous (CC vs. GG: p = 0.026; OR = 1.874, 95% CI = 1.079 to 3.257), heterozygous (GC vs. GG: p = 0.001; OR = 1.542, 95% CI = 1.273 to 1.868) and dominant model (CC + GC vs. GG: p = 0.001; OR = 1.631, 95% CI = 1.362 to 1.955). Similarly, increased TB risk was observed in subgroup analysis of Asian ethnicity. No publication bias was observed. These results suggested that *MIF* -173 G > C variant is a significant risk factor for TB in overall and in Asian populations, and can be used as prognostic marker for TB susceptibility.

## Introduction

*Mycobacterium tuberculosis* (*M.tb*) is the responsible microorganism of tuberculosis (TB) that still remains a second leading cause of death from any infectious disease worldwide. It is estimated that ~2 billion people are affected with *M.tb*, however only 5–10% of the infected population develop the active form of the disease^1^. The development of TB is affected by many factors including *M.tb* virulence, environmental factors, and various other risk factors comprising malnutrition, HIV infection, receipt of immunosuppressive therapy, and diabetes mellitus^2^. As mentioned earlier, only minority of the infected individuals actually develops clinical disease, this highlights an increased interest in understanding the role of genetic background of *M. tuberculosis* in infection outcome. Host genetic factors identified, why some people are more or less susceptible to TB infection. Several lines of evidences, including twin studies^3^, genome-wide linkage studies^4^, and lately reported genome-wide association studies (GWAS)^5,6^ have proven that host genetics strongly influences the susceptibility of TB. Hence, understanding the contribution of host genetic factor(s) in the development of TB infection could be proven as an extremely valuable tool in clinical and medicine research, especially because the susceptibility to an infectious agent lies at least partly hidden or masked in inborn errors or immune response^7^.

Human macrophage migration inhibitory factor (*MIF*) gene is located on chromosome 22q11.2 and encodes a multifunctional cytokine (MIF) that contributes to innate and adaptive immune responses against the pathogenic infection^8^. MIF is also known as a soluble factor produced by T-lymphocytes that can inhibit the migration and promote the aggregation of macrophages at the sites of local inflammation or infection^9^. In addition, MIF plays an important role in the regulation of the Th1/Th2 balance in the host’s inflammatory reaction and immune responses^10^. Two major polymorphisms are found in *MIF* gene, a functional variant (-173 G > C, rs755622) present within the 5′ promoter region, located at the position -173 involves a G > C substitution, appears to affect promoter activity in a cell-type dependent manner^11^. Previous clinical studies reported higher level of MIF in the serum of TB patients in comparison with the healthy controls^12^. Since, MIF protein appears to play a key role in mediating a wide variety of immune responses against the invading pathogens, for e.g., *M.tb* in humans. A large number of researchers performed genetic association studies to correlate *MIF* -173 G > C polymorphism with TB infection development^13–18^. However, the results from the published studies of -173 G > C gene polymorphism and TB susceptibility are inconsistent and inconclusive. The inconsistency in the results across many of the case-control studies could possibly because of lower sample size and individual studies. Furthermore, as the immune interactions determine TB susceptibility and severity, combined studies may give more meaningful information than a single, independent/individual study in determining the outcome of TB infection. Nowadays, meta-analysis, a statistical tool is used to pool the results from the individual studies to increase the sample size and provides more robust as well as reliable conclusion^19^. Therefore, a meta-analysis was carried out to evaluate the precise relationship between *MIF* -173 G > C genetic variant and the risk of developing TB, and its ethnicity based effect on TB infection. In this study, the quality of the included studies was checked by performing Newcastle Ottawa Scale (NOS) analysis. Additionally, publication bias and random errors (type-I statistical errors) occurred due to sparse data were minimized by performing Trial Sequential Analysis (TSA) for quantifying the statistical reliability of the data included in the cumulative meta-analysis with the threshold of statistical significance. This is the primary meta-analysis dealing with the precise appraisal of the association between the *MIF* -173 G > C gene polymorphism and the risk of TB.

## Results

### Literature search strategy

**Figure 1:**
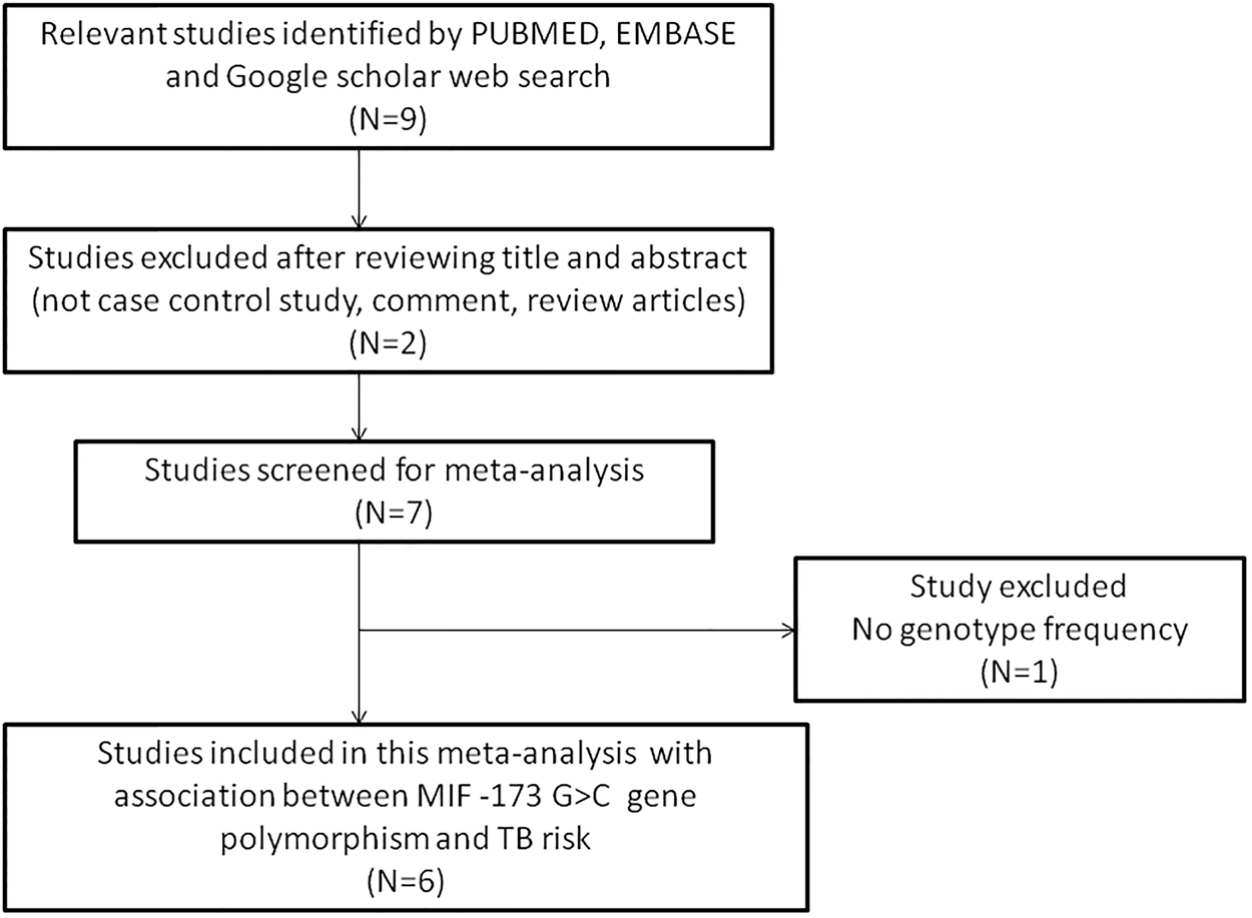
PRISMA 2009 Flow-Diagram showing the identification and selection process (inclusion/exclusion) of the eligible studies for the present meta-analysis.

According to the literature search strategy applied in this study, as stated in the methodology section, six articles^13–18^ were found relevant showing *MIF* -173 G > C polymorphism and occurrence of TB, and found eligible for inclusion in the current meta-analysis. All the retrieved research articles were evaluated carefully by examining their titles (headlines) and abstracts/summaries. Complete texts of the potentially germane research articles were further tested for their aptness for inclusion in the present meta-analysis. Studies either showing *MIF* genetic variant to predict survival in TB patients and/or considering *MIF* gene polymorphisms as indicators for response to therapy were disqualified straightaway. Likewise, research articles reporting *MIF* mRNA levels or protein expression or relevant reviews were omitted too. In this study, we included only case-control or cohort design based reports mentioning the frequency of all the three genotypes. Besides the database search, the references available in the retrieved studies were also examined for other probable articles. The major characteristics of all the six studies incorporated in this pooled study, i.e., distributions of genotypes, minor allele frequency (MAF) in the controls and cases, have been shown in Tables 1 and 2. The needful information related to the selection of the pertinent studies has been given in Fig. 1 (PRISMA 2009 Flow Diagram). All the six studies were assessed for their quality score as prescribed by the Newcastle Ottawa scale and most of the studies (80%) scored 5 or more stars and indicated moderate to good quality (Table 3).

**Table 1:** Main characteristics of all the six studies of *MIF* -173 G > C gene polymorphism and TB risk included in this meta-analysis.

First author [REF]	Country	Ethnicity	Type of Study	Controls	Cases	Type	Methods
Liu *et al*.^13^	China	Asian	HB	100	200	PTB	PCR-RFLP
Wang *et al*. ^14^	China	Asian		110	110	Spinal	PCR-RFLP
Hashemi *et al*.^15^	Iran	Asian	PB	142	161	PTB	PCR-RFLP
Li *et al*.^16^	China	Asian	HB	215	245	TB	PCR-RFLP
Sadki *et al*.^17^	Morocco	African	HB	154	123	PTB	TaqMan
Gomez *et al*.^18^	Colombia	Caucasian	HB	235	230	PTB	Sequencing

**Table 2:** Genotypic distribution of *MIF* -173 G > C gene polymorphism included in this meta-analysis.

First author [REF]	Controls				Cases				HWE
	Genotype			Minor allele	Genotype			Minor allele	
	GG	GC	CC	MAF	GG	GC	CC	MAF	P value
Liu *et al*.^13^	57	37	6	0.245	80	116	4	0.310	0.998
Wang *et al*.^14^	72	32	6	0.200	57	40	13	0.300	0.340
Hashemi *et al*.^15^	105	32	5	0.147	99	53	9	0.220	0.207
Li *et al*.^16^	168	61	16	0.189	109	74	32	0.320	0.002
Sadki *et al*.^17^	82	63	9	0.262	57	45	21	0.353	0.492
Gomez *et al*.^18^	133	86	16	0.251	120	95	15	0.271	0.680

**Table 3:** Quality assessment conducted according to the Newcastle-Ottawa Scale for all the six studies included in this meta-analysis.

First author [REF]	Quality indicators		
	Selection	Comparability	Exposure
Liu *et al*.^13^	***	*	**
Wang *et al*.^14^	****	*	**
Hashemi *et al*.^15^	****	*	*
Li *et al*.^16^	****	*	**
Sadki *et al*.^17^	***	*	*
Gomez *et al*.^18^	***	**	*

Note: On assessing the quality of the included studies using the Newcastle-Ottawa Scale, all the studies scored five stars or more which indicates no bias.

### Diagnosis of publication bias

In order to evaluate the publication bias among the incorporated studies, Begg’s funnel plot and Egger’s test were carried out in this meta-analysis. The appearance of the shape of funnel plots and the outcomes of Egger’s test have not shown the indication of publication bias in all the five genetic models (Table 4, Supplementary Information: Figure SI1).

**Table 4:** Statistics to test publication bias and heterogeneity in this meta-analysis for *MIF* -173 G > C gene polymorphism: Overall risk.

Comparisons	Egger’s regression analysis			Heterogeneity analysis			Model used for the meta-analysis
	Intercept	95% Confidence Interval	P value	Q value	P_heterogeneity_	I^2^ (%)	
C Vs G	1.75	−8.62 to 12.14	0.66	8.32	0.139	39.92	Fixed
CC Vs GG	−2.54	−9.75 to 4.66	0.38	11.08	0.050	54.91	Fixed
GC Vs GG	1.48	−8.72 to 11.69	0.707	6.99	0.221	28.51	Fixed
CC + GC Vs GG	1.76	−7.60 to 11.13	0.629	6.22	0.285	19.66	Fixed
CC Vs GC + GG	−2.92	−10.53 to 4.68	0.34	13.21	0.021	62.174	Random

### Evaluation of heterogeneity

In order to check heterogeneity among the included studies, Q-test and I^2^ statistics were used. Heterogeneity was not observed in all the four studied genetic models. However, heterogeneity was observed in one genetic model CC vs GC + GG. Thus, random effects model was used for synthesizing the data (Table 4).

### Association of *MIF* -173 G > C gene polymorphism and TB susceptibility

**Figure 2:**
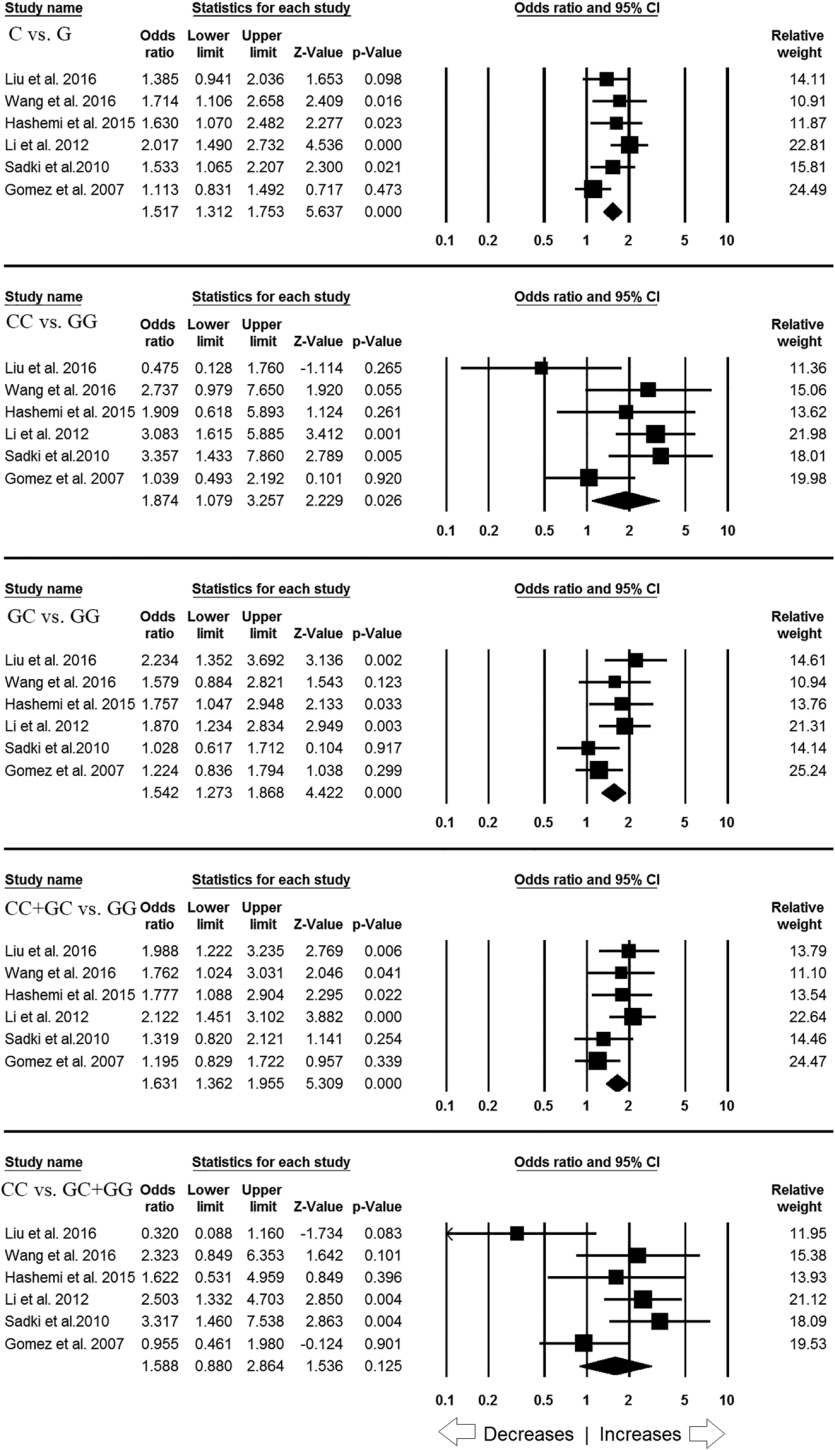
Forest plot of ORs with 95% CI of TB risk associated with the *MIF* -173 G > C gene polymorphism for overall population. Note: Black square represents the value of OR and the size of the square indicates the inverse proportion relative to its variance. Horizontal line is the 95% CI of OR.

All the six studies collectively produced 1069 confirmed TB cases and 956 healthy subjects. All the subjects were examined for the association between *MIF* -173 G > C polymorphism and overall TB risk. Overall pooled analysis suggested increased risk between *MIF* -173 G > C polymorphism and overall TB susceptibility in four genetic models, i.e., allelic (C vs G: p = 0.001; OR = 1.517, 95% CI = 1.312 to 1.753), homozygous (CC vs GG: p = 0.026; OR = 1.874, 95% CI = 1.079 to 3.257), heterozygous (GC vs GG: p = 0.001; OR = 1.542, 95% CI = 1.273 to 1.868), and dominant model (CC + GC vs GG: p = 0.001; OR = 1.631, 95% CI = 1.362 to 1.955). However, recessive (CC vs GC + GG: p = 0.125; OR = 1.588, 95% CI = 0.880 to 2.864) model did not display any risk between *MIF* -173 G > C polymorphism and overall TB risk (Fig. 2).

### Association of *MIF* -173 G > C gene variant & TB risk in Asian population

**Figure 3:**
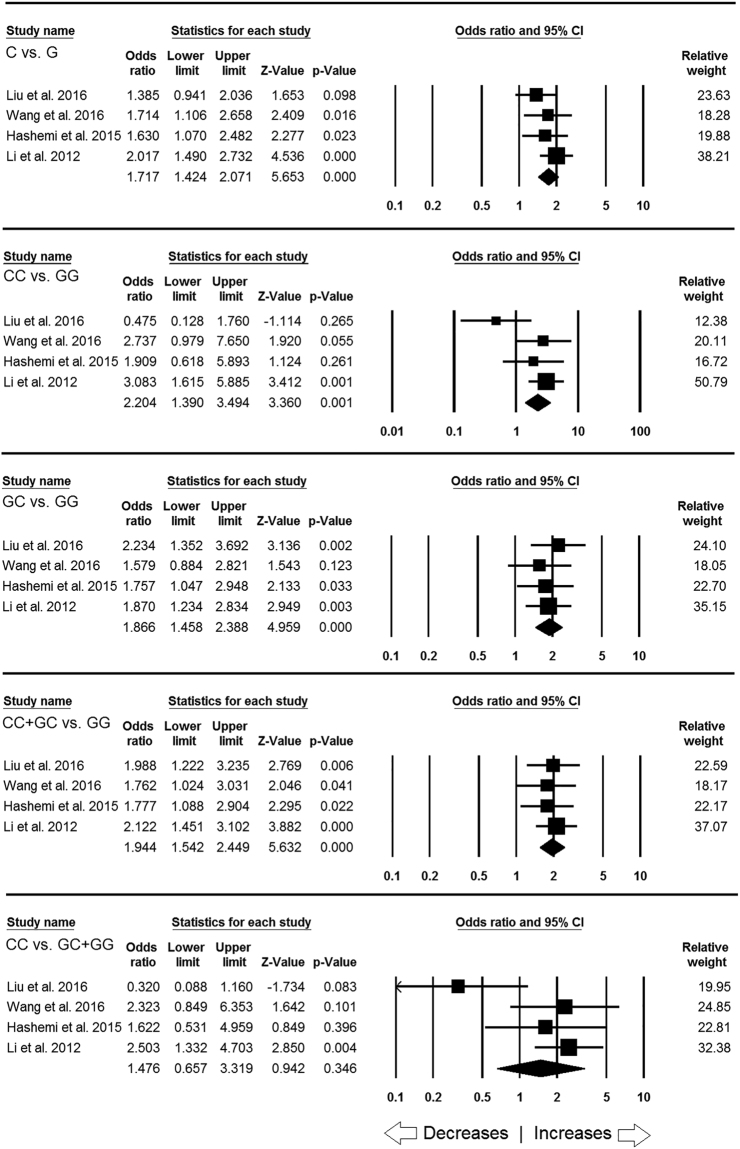
Forest plot of ORs with 95% CI of TB risk associated with the *MIF* -173 G > C gene polymorphism for Asian subgroup population. Note: Black square represents the value of OR and the size of the square indicates the inverse proportion relative to its variance. Horizontal line is the 95% CI of OR.

In sub-group analysis of Asian ethnicity population, four studies with a total number of 716 TB cases and 567 controls were included. Heterogeneity was not observed in all the four studied genetic models. However, heterogeneity was observed in recessive genetic model (Table 5). Also, we did not find any publication bias (Table 5; Figure SI2). After combining the all four studies, we observed that allelic (C vs G: p = 0.001; OR = 1.717, 95% CI = 1.424 to 2.071), homozygous (CC vs. GG: p = 0.001; OR = 2.204, 95% CI = 1.390 to 3.494), heterozygous (GC vs. GG: p = 0.001; OR = 1.866, 95% CI = 1.458 to 2.388) and dominant (CC + GC vs. GG: p = 0.001; OR = 1.944, 95% CI = 1.542 to 2.449) genetic models demonstrated increased risk of developing TB. Whereas, no TB risk was revealed by recessive genetic model (CC vs. GC + GG: p = 0.346; OR = 1.476, 95% CI = 0.657 to 3.319) (Fig. 3).

**Table 5:** Statistics to test publication bias and heterogeneity in this meta-analysis for *MIF* -173 G > C gene polymorphism: Asian population.

Comparisons	Egger’s regression analysis			Heterogeneity analysis			Model used for the meta-analysis
	Intercept	95% Confidence Interval	P value	Q-value	P_heterogeneity_	I^2^ (%)	
C Vs G	−3.42	−15.06 to 8.22	0.33	2.34	0.505	0.001	Fixed
CC Vs GG	−3.64	−12.28 to 5.00	0.21	6.53	0.088	54.12	Fixed
GC Vs GG	−1.42	−12.18 to 9.33	0.62	0.86	0.834	0.00	Fixed
CC + GC Vs GG	−2.23	−5.62 to 1.14	0.10	0.46	0.926	0.00	Fixed
CC Vs GC + GG	−3.83	−14.04 to 6.37	0.24	8.23	0.041	63.56	Random

### Sensitivity analysis

To evaluate the effect of individual studies for the risk of overall TB, we performed leave-one-out sensitivity analysis and recomputed the pooled ORs. The estimated pooled ORs calculated after excluding a single study did not show any differences from the primary values of overall and Asian risk of TB. This suggests that the results of this meta-analysis were stable and robust (Figures SI3 and SI4).

### Trial Sequential Analysis (TSA) of *MIF* -173 G > C gene polymorphism

**Figure 4:**
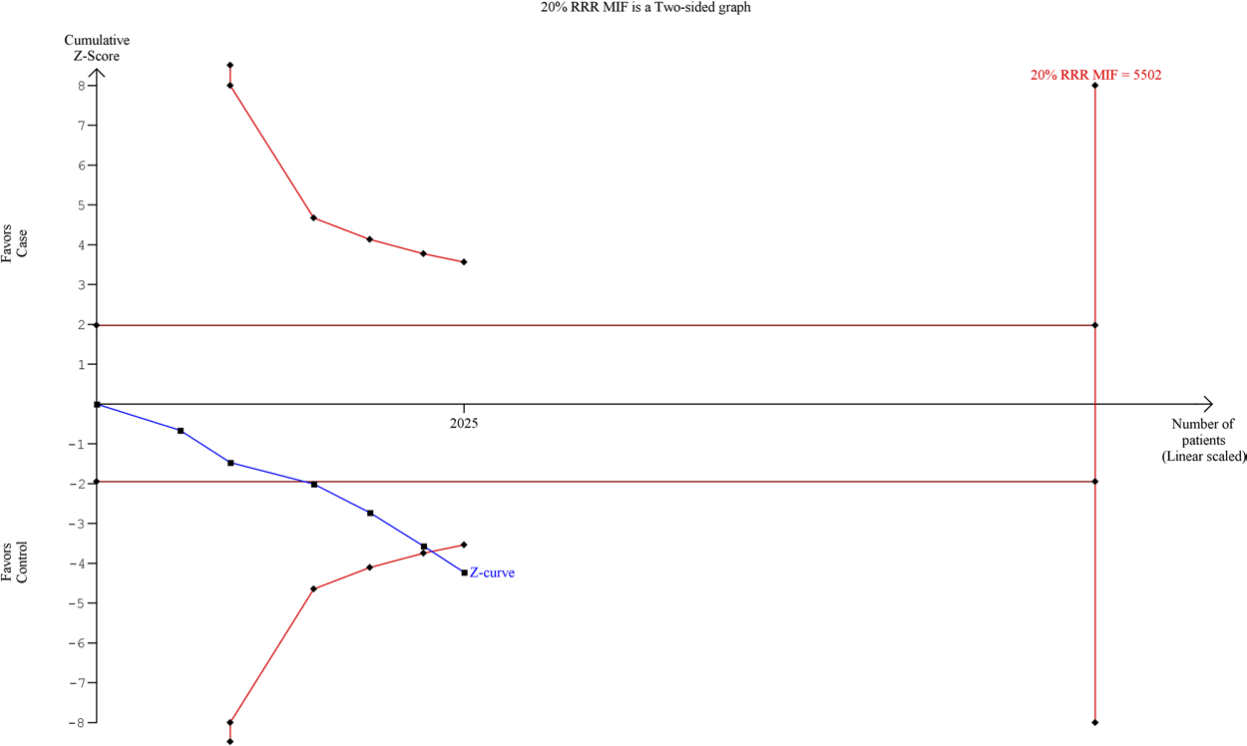
Trial sequence analysis of all the studies on *MIF* -173 G > C gene polymorphism based on dominant genetic model: Overall TB risk.

**Figure 5:**
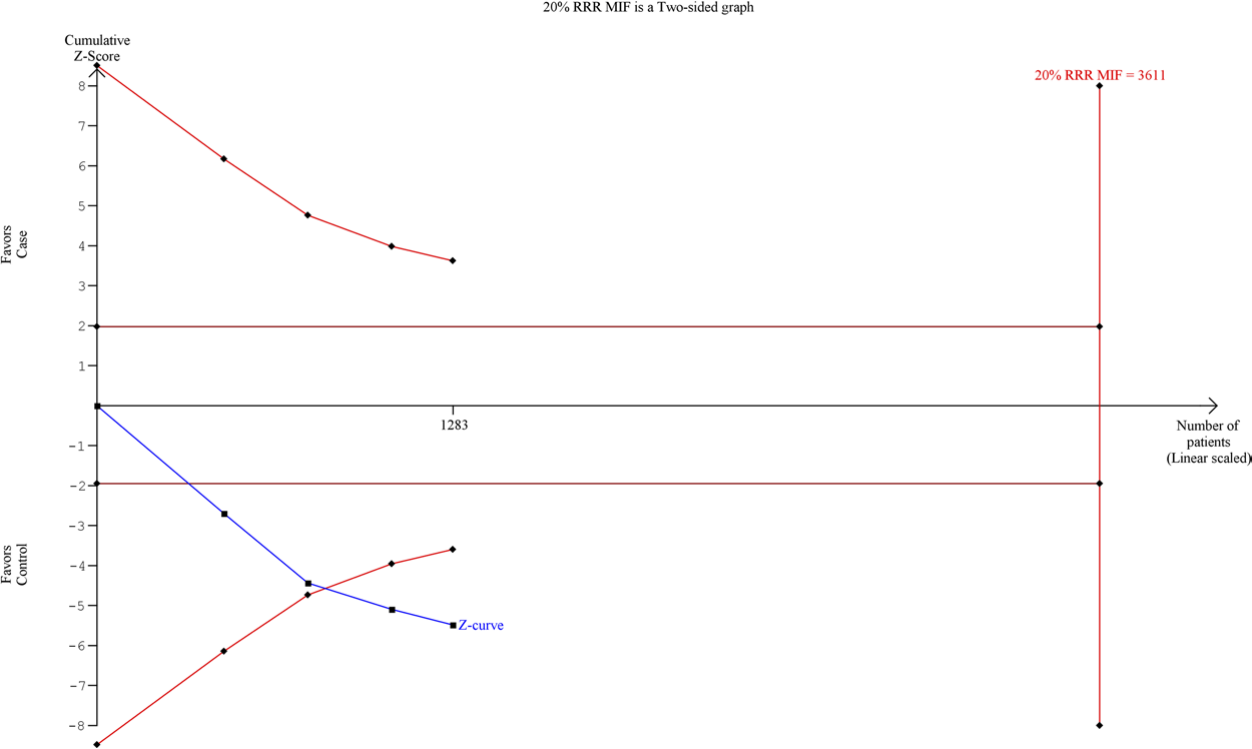
Trial sequence analysis of all the studies on *MIF* -173 G > C gene polymorphism based on dominant genetic model: TB risk in Asian population.

By employing TSA (taking the data of the dominant model), the cumulative Z curve crossed with TSA monitoring boundary confirmed that this polymorphism is associated with an increased risk of TB and further relevant trials are unnecessary (Fig. 4). Similar result was observed, when we performed the sub-group analysis (Asian) based on the ethnicity, TSA confirmed that *MIF* -173 G > C gene polymorphism is significantly associated with TB risk and further relevant trials are unnecessary (Fig. 5).

### Re-sampling results

The bootstrap re-sampling method was adopted to check the robustness of the findings of the present meta-analysis, and the relevant results have been given in the Supplementary Information (Microsoft Excel File SI5: Re-sampling analysis results of overall risk; Microsoft Excel File SI6: Re-sampling analysis results of Asian population) files provided with this manuscript. A total of 1000 re-sampling groups were created for both, overall and Asian ethnic groups that yielded 2025000 and 1283000 subjects, respectively. Re-sampling statistics of overall analysis revealed significant association of *MIF* -173 G > C (rs755622) polymorphism with TB susceptibility. The distribution of odds ratios, 95% CI, odds ratio and p-values for all the five genetic models (dominant, recessive, homozygous comparison, heterozygous comparison and allelic contrast) of overall analysis (2025000 subjects) have been shown in Table 6. Re-sampling statistics was also adopted to evaluate the association of *MIF* -173 G > C (rs755622) polymorphism with TB risk in Asian ethnicity (1283000 subjects). Similar to the re-sampling results of overall analysis, it was found that *MIF* -173 G > C (rs755622) polymorphism is also linked with TB susceptibility (Table 6).

**Table 6:** Re-sampling results of *MIF* -173 G > C gene polymorphism for overall and Asian population risk.

Population	Genetic model comparison	Odds Ratio range	Odds ratio	95% CI	p-value
Overall	C vs G	1.10 to 2.04	1.50	1.49 to 1.50	<0.0001
	CC vs GG	0.97 to 3.74	1.96	1.93 to 1.99	<0.0001
	GC vs GG	1.09 to 2.13	1.56	1.55 to 1.57	<0.0001
	CC + GC vs GG	1.21 to 2.24	1.62	1.61 to 1.63	<0.0001
	CC vs GC + GG	0.91 to 2.88	1.65	1.63 to 1.67	<0.0001
Asian	C vs G	1.23 to 2.01	1.57	1.56 to 1.58	<0.0001
	CC vs GG	0.89 to 3.11	1.57	1.55 to 1.60	<0.0001
	GC vs GG	1.34 to 2.61	1.96	1.94 to 1.97	<0.0001
	CC + GC vs GG	1.40 to 2.44	1.87	1.86 to 1.89	<0.0001
	CC vs GC + GG	0.74 to 2.26	1.23	1.22 to 1.25	<0.0001

## Discussion

As it is established that TB is a very complex, multi-factorial disease and environmental and host genetic factors are strongly involved in the development of TB infection^20^. Thus, prevention and control of TB is attracting more and more attention of the medical world. Identification of the host genes responsible for the susceptibility and resistance to TB may lead towards a better understanding of the pathogenesis of TB and management or even help in the treatment strategies. Evidences indicated that host genetic factors may play an important role in TB susceptibility that contains single nucleotide polymorphisms (SNPs) as a major factor^21^. Immune responses play a major role in the host defense against *M. tuberculosis* infection and can cause a broad spectrum of clinical manifestations^22,23^. Macrophages are the first line of the defense against *M.tb* infection. Invasive *M.tb* is eliminated or suppressed by phagosomal and lysosomal fusion, apoptosis induction, and free radicals^23^. The cytokines produced at the site of the disease after the interaction between T lymphocytes and infected macrophages are necessary for the pathogenesis of TB^24^.

*MIF* is an immunoregulatory cytokine that is expressed by T cells, macrophages and pulmonary epithelial cells, and has been considered as potential defense against the invading pathogen^25^. In addition, this gene has a key role in innate immune response against the bacteria and intracellular pathogens^26^. In the recent year, many researchers paid attention on the possible role of *MIF* gene in the innate immune system, pathogenesis of infection and inflammation^27,28^.

Considering the importance of *MIF* gene in view, previously many studies examined the possible link between *MIF* -173 G > C gene variant and the development of TB infection but failed to give concrete conclusion, and still the said relationship is controversial. Until now, there is no well-organized meta-analysis available that evaluated the precise association between *MIF -*173 G > C gene polymorphism and risk of TB infection development. Therefore, we conducted the present meta-analysis to precisely investigate this possible relationship. On the basis of six independent publications, our meta-analysis clearly produced statistical evidence that *MIF* -173 G > C variant is significantly associated with an increased risk of TB infection. As we know that host genetic factors are potentially responsible for the development of TB infection^20^. Hence, a single genetic polymorphism is normally inadequate to prophesized the susceptibility towards this dreadful disease. The important feature of this gene variant is that its occurrence may vary sufficiently among different ethnic populations or races. Therefore, ethnicity-wise sub-group analysis using stratification analysis was performed, which revealed that *MIF* -173 G > C is more prominent in the Asian subjects. Also, the precise association suggested by the current meta-analysis was confirmed by the TSA, which further escalated the conclusion that *MIF* -173 G > C polymorphism is correlated with an increased risk of TB. The polymorphism of the promoter region may alter the expression of mRNA and the level of protein. Earlier studies reported that *MIF* -173 C allele significantly increase the promoter activity of *MIF* gene^26^. Earlier, studies stated that circulating levels of MIF concentration were significantly higher in those affected with TB infection than in the healthy controls^12,13^. Promoter sequence analysis has deciphered that *MIF* -173 C allele creates a potential activator protein 4 transcription factor binding site, and the circulating levels of MIF are significantly vary among *MIF* -173 G > C genotypes^29^. MIF is also considered an essential component of the host antimicrobial alarm system and plays an important role in the stress response that promotes the proinflammatory functions of immune cells^30^. Although, it is worth mentioning that the immune interactions between the host and the molecular structure of *M.tb* are not solely depend upon one single factor or one single gene variant, but rather on many gene variants or gene-environment interactions.

Regardless of achieving some crucial findings, the present meta-analysis suffered with several limitations. First, we considered the research articles that were published only in the English language, which resulted into limited number of studies. The research studies published in other languages and probable unpublished articles were not included in this meta-analysis, which may cause publication bias. Second, in the stratification analysis by sub-group ethnicity-wise analysis, the numbers of individual studies are less that might have a small statistical power to detect the actual association. Third, the study Li *et al*.^16^ did not fit with the HWE test in the control group. The most probable reason for this variation is either the controls were not recruited from exactly the same genetic population as the TB patients or may controls suffered from other infectious diseases. But, after the omission of the study of Li *et al*.^16^ during the sensitivity analysis, it did not alter the conclusions observed in the meta-analysis. Fourth, the results presented here in this study were based on unadjusted estimates, as ORs in all the included studies were not adjusted by the same potential confounders, such as age, sex, and exposure. Fifth, some studies included in the present meta-analysis used PCR-RFLP whereas other studies adopted sequencing for the genotyping purpose. The basic difference between these two techniques is that, RFLP-PCR exploits variations in homologous DNA sequences and allows rapid detection of the mutation after genomic sequences PCR amplification followed by specific restriction enzyme digestion and visualization in stained agarose gel. Whereas, DNA sequencing is any chemical, enzymatic or technological procedure for determining the linear order of nucleotide bases in DNA, it is generally used to detect any mutation in the DNA sequence being analyzed and this method is often considered as the ‘gold standard’ for DNA sequence analysis. Both of these methods have their own limitations such as sensitivity, performance ease, and instrumentation constraints etc., that might affect the overall results in one or another way.

Notwithstanding above limitations, there are some advantages associated with this meta-analysis. First, the quality assessment of the selected studies was stringently performed by applying NOS scale, where most of the studies scored five or more stars in quality assessment score and indicated moderate to good quality of each study by clearly mentioning about the sample size, genotype, and inclusion criteria of confirmed TB patients and healthy controls. Second, in order to achieve more robust conclusion, publication bias analysis was done and the results did not show any publication bias. Third, we minimized the likelihood of bias by following a detailed protocol of study identification, statistical analysis and data selection. Fourth, sensitivity analysis, Trial sequential analysis, and re-sampling statistics further strengthened the conclusion of the present study.

In conclusion, this meta-analysis provides a strong evidence that *MIF* -173 G > C gene polymorphism is associated with the development of TB infection in overall and Asian population. *MIF* genotyping may offer the possibility of identifying individuals those are at a greater risk of TB. Overall, these results would help in understanding the role of this promoter variant in TB infection development and can aid in identifying new molecular targets by focusing on TB patients most likely to get benefit from the pharmacological targeting of *MIF*. Further well-designed and large sample studies are warranted to confirm the findings reported in this study. Additionally, studies investigating the effect of gene-environment interactions in relation with TB risk are also required.

## Materials and Methods

### Identification and suitability of the pertinent studies

A comprehensive and systematic search of the relevant studies based on the association between *MIF* -173 G > C (rs755622) gene polymorphism and the development of TB conducted on internet using online databases, i.e., PubMed (Medline), Google Scholar, and EMBASE to retrieve the compatible and peer reviewed research articles. The last search updated on February 2017. The pertinent study search was performed using the combination of the following keywords: ‘macrophage migration inhibitory factor OR *MIF* AND Tuberculosis OR TB susceptibility OR TB risk. Combination of single nucleotide polymorphism (SNP) OR genetic variant OR mutation OR genetic polymorphism of *MIF* -173 G > C AND/OR *MIF* rs755622 was also tried for the retrieval of the relevant studies. The search was focused on the studies that had been conducted in humans only. All the retrieve studies were evaluated by reading their titles (headlines) and abstracts/summaries, and all the published articles matching with the pre-set eligibility norms were retrieved for the present meta-analysis. We also did some manual search of the references listed in the retrieved articles for other eligible articles.

### Inclusion and exclusion criteria

In order to include the research articles in the current meta-analysis, retrieved studies had to fulfill all the pre-set criteria, (a) it must have evaluated the relationship between *MIF* -173 G > C gene polymorphism and susceptibility to TB, (b) adopted a case-control study design, (c) must recruited confirmed TB patients and healthy control subjects, (d) availability of genotype frequency in cases (confirmed TB patients) and controls to count the odds ratio (OR) and 95% confidence intervals, (e) must published in the English language. Likewise, some preset criteria for the study exclusion were also applied. The major reasons for the study exclusion were (i) not designed as a case-control study, (ii) reviews, and abstracts or overlapping studies, (iii) not reported the genotype frequencies or numbers in the studies. The schematic flow diagram of the selection of the pertinent studies for this meta-analysis following the pre-set including/excluding criteria is shown in Fig. 1: PRISMA 2009 Flow-Diagram.

### Data extraction

For every retrieved published study, the procedural data extraction was independently performed in replica copies by two independent (self-regulating) investigators (SAD & RKM) by following a standard validated protocol. In order to ratify the accuracy of the abstracted data, strict pre-set selection criteria were followed during the collection of the data by filling a standard data-collection form. The major characteristics summarized from the retrieved articles include the name of the first author, year of publication, the number of cases and controls, the country of origin, type of study, TB type, methods of genotyping and genotype frequencies for the cases and the controls. Cases pertaining to any type of disagreement or discrepancy in any of the items of the abstracted data from the selected articles were fully resolved by conducting many rounds of group discussions involving adjudicator (SH) to accomplish a final agreement.

### Quality score assessment by Newcastle-Ottawa Scale (NOS) criteria

The methodological quality evaluation of the selected studies was also performed separately by two independent investigators (SAD & RKM) by following the preset NOS criteria^31^. The NOS criteria predominantly comprised of three aspects: (i) subject selection: 0–4 points; (ii) comparability of subjects: 0–2 points; (ii) clinical outcomes: 0–3 points. The selected studies, which were awarded 5 stars or more, can be considered as moderate to high quality^32^. The quality assessment of the selected individual study was performed independently in duplicate by two investigators (as stated above SAD & RKM) and in case of any discord, it was resolved by a systematic discussion or expert consultation, if necessary with the help of adjudicator SH).

### Statistical analysis

The precise strength of the association of *MIF* -173 G > C gene variant with the chances of developing TB was assessed by using OR and 95% CI. The pooled ORs were computed for allele contrast, log-additive, recessive, and dominant genetic models^33^. Heterogeneity assumption between the studies across the eligible comparison was done by the chi-square based Q-test^34^. Heterogeneity was deliberated significant in case p-value < 0.05, to avoid underestimation of the presence of heterogeneity. A fixed effect model (if p > 0.05) or a random effect model (if p < 0.05)^35,36^ was applied for the pooling of the results. In addition, I^2^ statistics was used to efficiently check the heterogeneity among the chosen studies^37^. Hardy-Weinberg equilibrium (HWE) in the controls was computed by employing chi-square test. Additionally, funnel plot asymmetry was measured by Egger’s linear regression test (a type of linear-regression approach) that calculates the funnel plot asymmetry based on the natural logarithm scale of the ORs. The significance of the intercept was determined by the t-test, where p-value < 0.05 was deliberated as the symbol of significant statistical publication bias^38^. The entire statistical analysis involved in this meta-analysis was performed by using Comprehensive Meta-Analysis (CMA) version 2 software program (Biostat, NJ, USA). All the p-values were two sided and the statistical significance level was considered as p-value < 0.05 for this pooled analysis.

### Re-sampling statistics

As number of the studies included in this meta-analysis is small, a bootstrap re-sampling method was adopted by creating 1000 re-sampling groups followed by statistical analysis for possible association of *MIF* polymorphism (rs755622) with TB susceptibility^39,40^. The details of re-sampling program along with its results are shown in the Supplementary Information (Microsoft Excel File SI5 and Microsoft Excel File SI6). In the re-sampling program, Microsoft excel file data of the recruited studies (for overall association analysis) comprising 1069 confirmed TB patients and 956 healthy controls were coded as follows: number 1 for TB patients with GG genotype, 2 for TB patients with GC genotype, 3 for TB patients with CC genotype, 4 for healthy controls with GG type, 5 for controls with GC genotype, and 6 for healthy controls with CC genotype. For each re-sampling group, genotypes of 2025 samples were generated by bootstrap method and ORs were calculated for various genetic models. A total sum of 1000 re-sampling groups were created for robust and replicable results. Overall, ORs, 95% CI, range of ORs and p-values were calculated for all the five genetic models (Supplementary Information: Microsoft Excel File SI5). Furthermore, similar re-sampling analysis was applied for Asian cohort comprising 716 confirmed TB patients: 716 and 567 healthy controls (Supplementary Information: Microsoft Excel File SI6).

### Trial sequential analysis (TSA)

According to Cochrane handbook, meta-analyses are considered to be the best if all the eligible trials are included in the analysis, however, it may not be able to provide adequate and satisfactory evidence. As meta-analysis may leads to systematic errors (bias) or random errors (play of chance). In order to minimize these errors, a novel statistical analysis program, Trial Sequential Analysis (TSA) tool from Copenhagen Trial Unit, Center for Clinical Intervention Research, Denmark) has been introduced that estimates required information size and adjusts the threshold for the statistical significance and estimates the power of current conclusion^41–43^. If, a TSA monitoring boundary is crossed with Z curve before the required information size is reached, robust evidence might have been confirmed and further trials are unnecessary. In contrast, if the Z curve does not cross the monitoring boundaries and the required information size has not been reached, then it is necessary to continue doing the trials. Trial Sequential Analysis (version 0.9, http://www.ctu.dk/tsa/) was utilized for performing the present investigation.

## Electronic supplementary material

Supplementary Information SI1-SI4

Supplementary Information: Microsoft Excel File SI5

Supplementary Information: Microsoft Excel File SI6
