# Supplementary material for: MIF -173 G > C (rs755622) Gene Polymorphism Modulates Tuberculosis Risk: Evidence from a Meta-analysis and Trial Sequential Analysis
Source: Sci Rep. 2017 Dec 5;7:17003. doi: 10.1038/s41598-017-17308-y (PMC5717251; doi:10.1038/s41598-017-17308-y)
Supplement: Supplementary file 1 — Supplementary Information SI1-SI4 [file 41598_2017_17308_MOESM1_ESM.pdf]

Supplementary Information

MIF -173 G>C (rs755622) Gene Polymorphism Modulates Tuberculosis Risk: Evidence from a Meta-analysis and Trial Sequential Analysis

Authors' names in order of their authorship
Mohammed Y. Areeshi[1], Raju K. Mandal[1], Sajad A. Dar[1,2], Arshad Jawed[1], Mohd Wahid[1], Mohtashim Lohani[1], Aditya K Panda[3], B.N. Mishra[4], Naseem Akhter[5], Shafiul Haque[1]✉

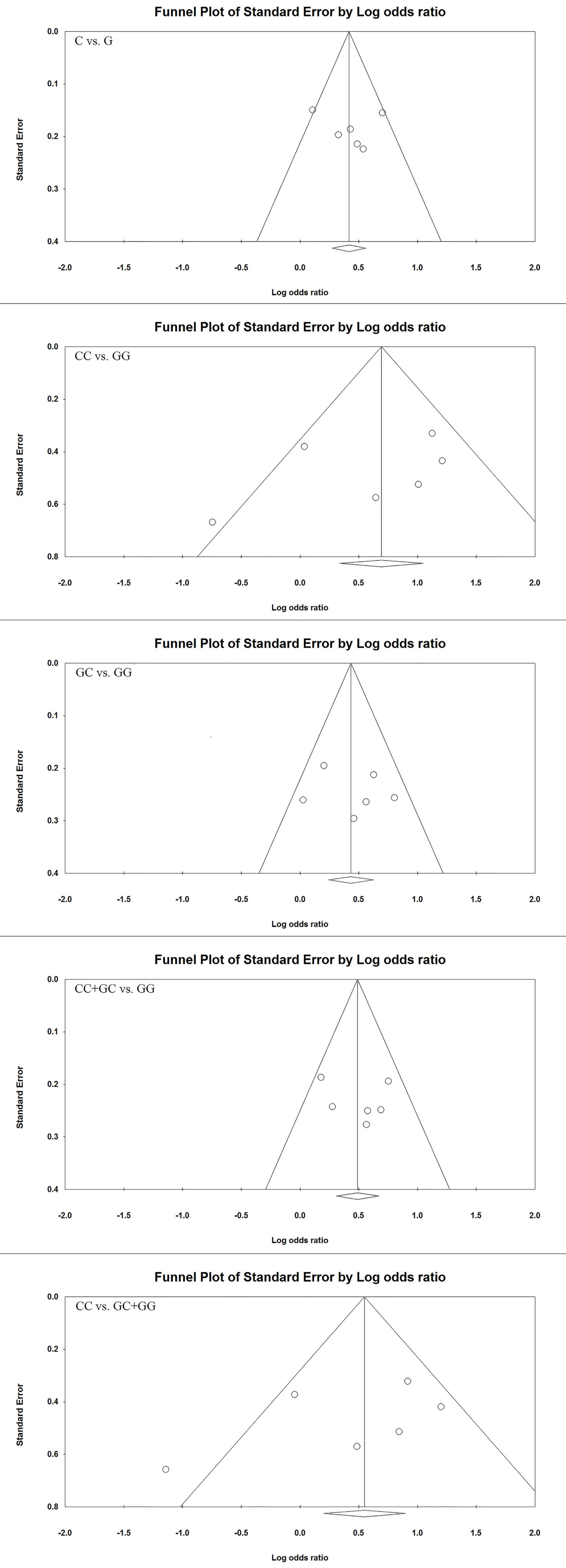

Figure SI1: Assessment of publication bias shown with Funnel plots in studies assaying odds of TB associated with the MIF -173 G>C gene polymorphism for overall analysis (Odds ratio against standard error in different genetic models).

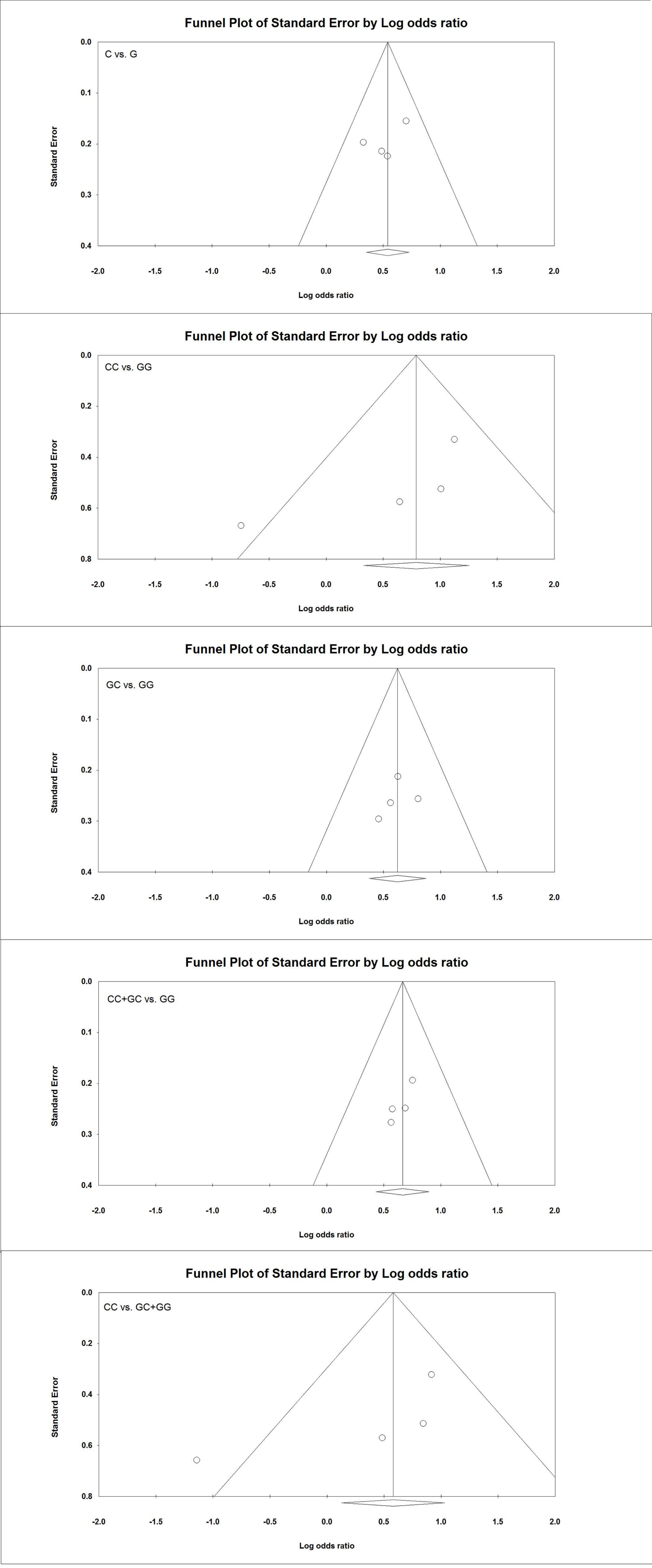

Figure SI2: Assessment of publication bias shown with Funnel plots in studies assaying odds of TB associated with the MIF -173 G>C gene polymorphism for sub-group (Asian ethnicity population) analysis (Odds ratio against standard error in different genetic models).

Study name — **Statistics with study removed** — **Odds ratio (95% CI) with study removed**

C vs. G

Study name	Point	Lower limit	Upper limit	Z-Value	p-Value
Liu et al. 2016	1.540	1.317	1.800	5.413	0.000
Wang et al. 2016	1.494	1.282	1.742	5.130	0.000
Hashemi et al. 2015	1.502	1.287	1.753	5.169	0.000
Li et al. 2012	1.394	1.182	1.644	3.951	0.000
Sadki et al.2010	1.514	1.293	1.773	5.147	0.000
Gomez et al. 2007	1.677	1.419	1.981	6.079	0.000
	1.517	1.312	1.753	5.637	0.000

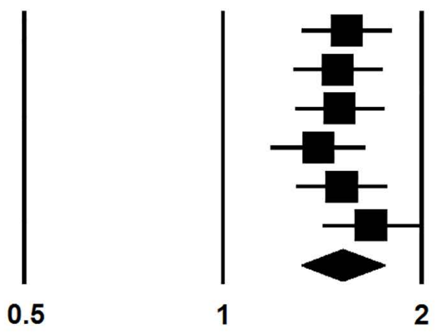

Study name — **Statistics with study removed** — **Odds ratio (95% CI) with study removed**

CC vs. GG

Study name	Point	Lower limit	Upper limit	Z-Value	p-Value
Liu et al. 2016	2.235	1.399	3.570	3.364	0.001
Wang et al. 2016	1.731	0.903	3.317	1.652	0.098
Hashemi et al. 2015	1.846	0.962	3.542	1.845	0.065
Li et al. 2012	1.626	0.852	3.100	1.475	0.140
Sadki et al.2010	1.641	0.871	3.091	1.532	0.125
Gomez et al. 2007	2.208	1.245	3.916	2.709	0.007
	1.874	1.079	3.257	2.229	0.026

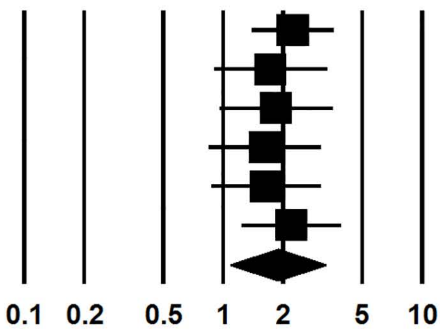

Study name — **Statistics with study removed** — **Odds ratio (95% CI) with study removed**

GC vs. GG

Study name	Point	Lower limit	Upper limit	Z-Value	p-Value
Liu et al. 2016	1.447	1.176	1.782	3.488	0.000
Wang et al. 2016	1.538	1.255	1.885	4.145	0.000
Hashemi et al. 2015	1.510	1.228	1.857	3.910	0.000
Li et al. 2012	1.464	1.179	1.817	3.450	0.001
Sadki et al.2010	1.649	1.340	2.028	4.730	0.000
Gomez et al. 2007	1.667	1.335	2.082	4.511	0.000
	1.542	1.273	1.868	4.422	0.000

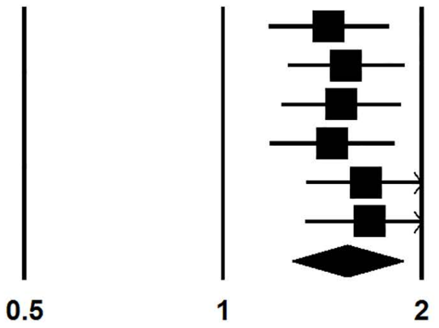

Study name — **Statistics with study removed** — **Odds ratio (95% CI) with study removed**

CC+GC vs GG

Study name	Point	Lower limit	Upper limit	Z-Value	p-Value
Liu et al. 2016	1.581	1.301	1.920	4.610	0.000
Wang et al. 2016	1.616	1.334	1.957	4.908	0.000
Hashemi et al. 2015	1.610	1.325	1.955	4.801	0.000
Li et al. 2012	1.511	1.230	1.855	3.936	0.000
Sadki et al.2010	1.691	1.391	2.056	5.271	0.000
Gomez et al. 2007	1.804	1.466	2.221	5.564	0.000
	1.631	1.362	1.955	5.309	0.000

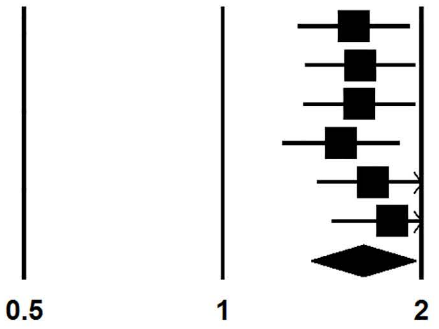

Study name — **Statistics with study removed** — **Odds ratio (95% CI) with study removed**

CC vs. GC+GG

Study name	Point	Lower limit	Upper limit	Z-Value	p-Value
Liu et al. 2016	1.973	1.248	3.119	2.908	0.004
Wang et al. 2016	1.461	0.728	2.935	1.066	0.286
Hashemi et al. 2015	1.562	0.781	3.126	1.261	0.207
Li et al. 2012	1.393	0.675	2.875	0.897	0.370
Sadki et al.2010	1.350	0.704	2.588	0.903	0.367
Gomez et al. 2007	1.797	0.931	3.466	1.748	0.081
	1.588	0.880	2.864	1.536	0.125

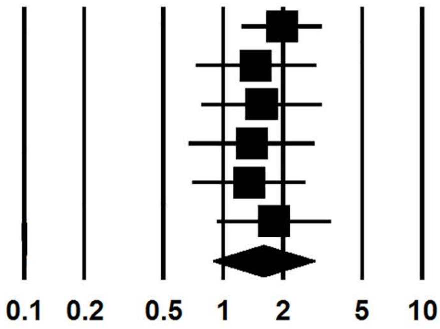

Figure SI3: Sensitivity analysis of MIF -173 G>C gene polymorphism with overall TB risk to evaluate the influence of each individual study on the pooled OR by deleting one single study each time for overall analysis (for all the genetic models) Black square represents the value of OR and the size of the square indicates the inverse proportion relative to its variance. Horizontal line is the 95% CI of OR.

C vs. G

Study name	Point	Lower limit	Upper limit	Z-Value	p-Value
Liu et al. 2016	1.836	1.481	2.275	5.549	0.000
Wang et al. 2016	1.718	1.396	2.114	5.114	0.000
Hashemi et al. 2015	1.740	1.411	2.145	5.181	0.000
Li et al. 2012	1.554	1.225	1.973	3.625	0.000
	1.717	1.424	2.071	5.653	0.000

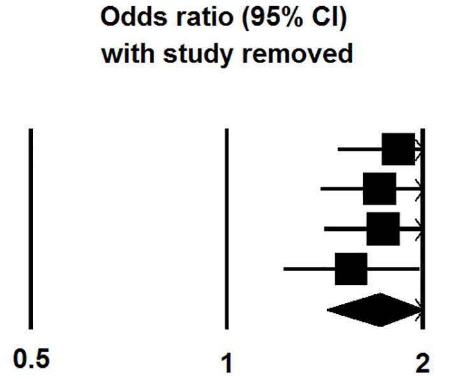

CC vs. GG

Study name	Point	Lower limit	Upper limit	Z-Value	p-Value
Liu et al. 2016	2.737	1.673	4.479	4.008	0.000
Wang et al. 2016	2.087	1.246	3.495	2.796	0.005
Hashemi et al. 2015	2.268	1.369	3.759	3.178	0.001
Li et al. 2012	1.559	0.808	3.007	1.324	0.186
	2.204	1.390	3.494	3.360	0.001

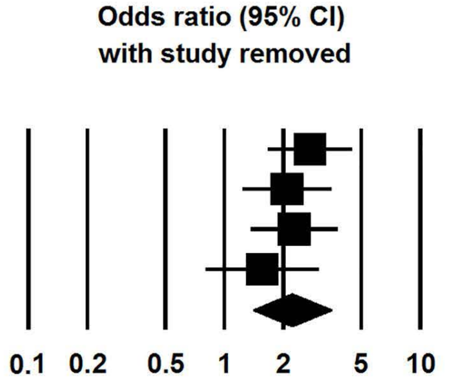

GC vs. GG

Study name	Point	Lower limit	Upper limit	Z-Value	p-Value
Liu et al. 2016	1.763	1.328	2.340	3.926	0.000
Wang et al. 2016	1.936	1.475	2.543	4.755	0.000
Hashemi et al. 2015	1.900	1.435	2.515	4.485	0.000
Li et al. 2012	1.865	1.373	2.533	3.988	0.000
	1.866	1.458	2.388	4.959	0.000

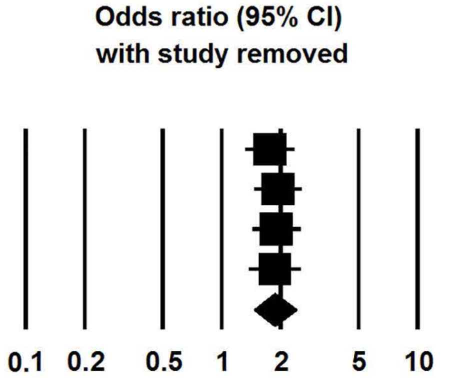

CC+GC vs. GG

Study name	Point	Lower limit	Upper limit	Z-Value	p-Value
Liu et al. 2016	1.931	1.484	2.511	4.906	0.000
Wang et al. 2016	1.986	1.538	2.565	5.262	0.000
Hashemi et al. 2015	1.994	1.534	2.591	5.159	0.000
Li et al. 2012	1.846	1.379	2.470	4.120	0.000
	1.944	1.542	2.449	5.632	0.000

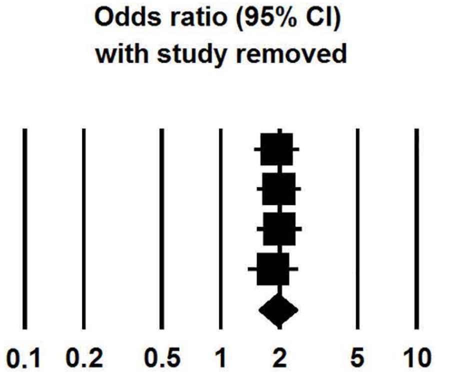

CC vs. GC+GG

Study name	Point	Lower limit	Upper limit	Z-Value	p-Value
Liu et al. 2016	2.269	1.401	3.675	3.332	0.001
Wang et al. 2016	1.209	0.386	3.789	0.326	0.744
Hashemi et al. 2015	1.372	0.450	4.182	0.557	0.578
Li et al. 2012	1.127	0.364	3.486	0.207	0.836
	1.476	0.657	3.319	0.942	0.346

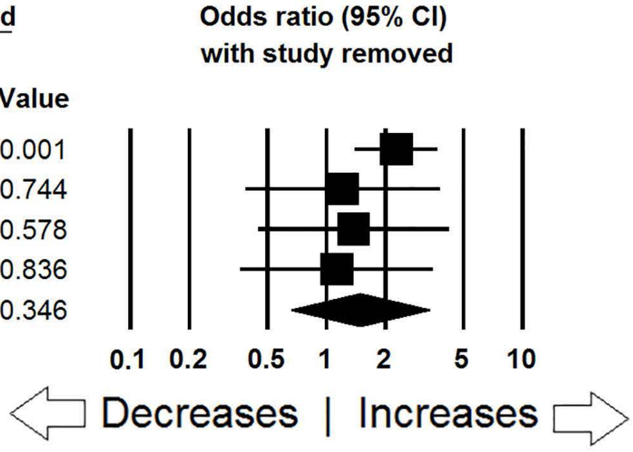

Figure SI4: Sensitivity analysis of MIF -173 G>C gene polymorphism with TB risk among Asian population to evaluate the influence of each individual study on the pooled OR by deleting one single study each time for subgroup analysis (for all the genetic models). Black square represents the value of OR and the size of the square indicates the inverse proportion relative to its variance. Horizontal line is the 95% CI of OR.
